# Language and cognition—joint acquisition, dual hierarchy, and emotional prosody

**DOI:** 10.3389/fnbeh.2013.00123

**Published:** 2013-09-19

**Authors:** Leonid Perlovsky

**Affiliations:** The AFRL and Athinoula A. Martinos Center for Biomedical Imaging, Harvard UniversityCharlestown, MA, USA

**Keywords:** language, cognition, acquisition, dual hierarchy, prosody, emotion

## Function of language and cognition in thinking

Do we think with language, or is it just a communication device used for expression of completed thoughts? What is a difference between language and cognition? Chomsky (1995) suggested that these two abilities are separate and independent. Cognitive linguistics emphasizes a single mechanism for both (Croft and Cruse, 2004). Evolutionary linguistics considers the process of transferring language from one generation to the next one (Cangelosi and Parisi, 2002; Christiansen and Kirby, 2003; Hurford, 2008). This process is a “bottleneck” that forms the language. Brighton et al. (2005) demonstrated emergence of compositional language due to this bottleneck. Still, none of these approaches resulted in a computational theory explaining how humans acquire language and cognition. Here I discuss a computational model overcoming previous difficulties and based on a hypothesis that language and cognition are two separate and closely integrated abilities. I identify their functions and discuss why human thinking ability requires both language and cognition.

Among fundamental mechanisms of cognition are mental representations, memories of objects and events (Perlovsky, 2001, 2006a). The surrounding world is understood by matching mental representations to patterns in sensor signals. However, mathematical modeling of this process since the 1950s met with difficulties. The first difficulty is related to a need to consider combinations of sensor signals, objects, and events. The number of combinations is very large and even a limited number of signals or objects form a very large number of combinations, exceeding all interactions of all elementary particles in a lifetime of the Universe (Perlovsky, 1998). This is known as combinatorial complexity, CC. This difficulty in modeling the mind has been overcome by dynamic logic (Perlovsky, 2001, 2006a,b, 2007a; Perlovsky et al., 2011). Whereas classical logic considers static statements such as “this is a chair,” dynamic logic models processes from vague to crisp representations. These processes do not need to consider combinations, an initial vague state of a “chair” matches any object in the field of view, and at the end of the process it matches the chair actually present, without CC.

The second difficulty is similar still even more complex. It is related to the fact that “events” and “situations” in the world do not necessarily exist “ready for cognition.” There are many combinations of percepts and objects, a near infinity, events and situations important for understanding and learning have to be separated from those that are just random collections of meaningless percepts or random objects (Perlovsky and Ilin, 2012). Events and situations recognized by non-human animals are very limited compared to human abilities to differentiate events in the world. Human cognitive abilities acquire their power due to language. Language is “easier” to learn than cognitive representations. Language representations: words, phrases exist in the surrounding language “ready made,” created during millennia of cultural evolution. Therefore, language could be learned without much real-life experience; only interactions with language speakers are required. Every child learns language early in life before acquiring full cognitive understanding of events and their cognitive meanings. Thus, language is learned early in life with only limited cognitive understanding of the world (Perlovsky, 2009a, 2012c). Cognitive representations of situations and abstract concepts initially exist in vague states. Throughout the rest of life, language guides acquisition of cognitive representations from experience. Vague cognitive representations become more crisp and concrete. Thinking involves both language and cognition, and as we discuss later thinking about abstract ideas usually involves language more than cognition, not too different from thinking by children.

## The dual hierarchy

**Figure 1:**
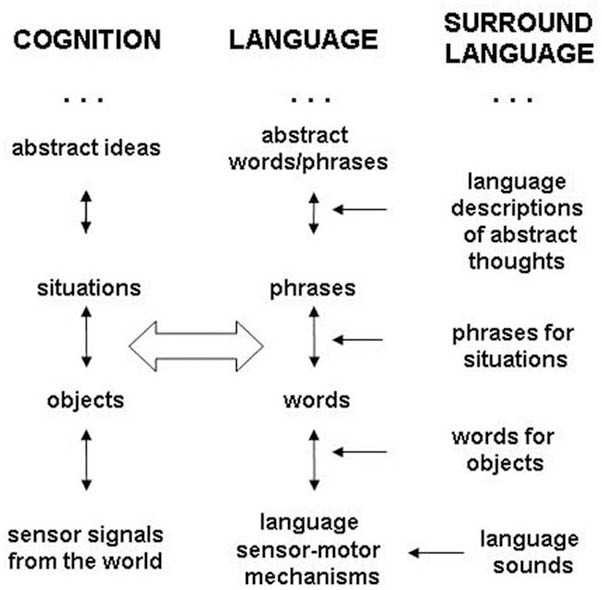
**The dual hierarchy.** Language and cognition are organized into approximate dual hierarchy. Learning language is grounded in the surrounding language throughout the hierarchy. Cognitive hierarchy is grounded in experience only at the very “bottom.”

Cognitive representations are organized in mind in an approximate hierarchy (Grossberg, 1988) from sensor-motor percepts near “bottom,” to objects “higher up,” to situations, and to still more abstract cognitive representations. Language representations are organized in a parallel hierarchy from sounds, and words for objects and situations, to phrases, and to more abstract language representations. Our previous discussion can be described by an integrated mathematical model of language and cognition forming a dual hierarchy (Perlovsky, 2009a), as illustrated in Figure 1. Neural evidence suggests that the hierarchy is approximate, not as definite as shown in this figure.

Hierarchical organization of cognition and related brain structures are reviewed in (Badre, 2008). In particular, anterior-posterior axis corresponds to a gradient of abstract-concrete cortex functions. Hierarchical organization of language functions is also well established. However, hierarchical organization of language does not correspond to a particular spatial axis in the brain, it is distributed (Price, 2012). Therefore, the dual hierarchy in Figure 1 is a functional hierarchy not organized along a spatial axis in the brain as in this figure. A fundamental aspect of acquiring mental representations is interaction between higher and lower layer representations (top and bottom layers). In this interaction a lower layer representations are organized in more abstract and general concept-representations at a higher layer. These interactions are referred to as bottom-up and top-down signals (BU and TD) indicated in Figure 1 by vertical arrows.

Mathematical model of the dual hierarchy is described in Perlovsky (2009a, 2012c) and Perlovsky and Ilin (2010, 2012). This model explains many facts about thinking, language, and cognition, which has remained unexplainable and would be considered mysteries, if not so commonplace.

The dual model makes a number of experimentally testable predictions. (1) It explains functions of language and cognition in thinking: cognitive representations model surrounding world, relations between objects, events, and abstract concepts. Language stores culturally accumulated knowledge about the world, yet language is not directly connected to objects, events, and situations in the world. Language guides acquisition of cognitive representations from random percepts and experiences, according to what is considered worth learning and understanding in culture. Events that are not described in language are likely not even noticed or perceived in cognition. (2) Whereas language is acquired early in life, acquiring cognition takes a lifetime. The reason is that language representations exist in surrounding language “ready-made,” acquisition of language requires only interaction with language speakers, but does not require much life experience. Cognition on the opposite requires life experience. (3) This is the reason why abstract words excite only language regions of brain, whereas concrete words excite also cognitive regions (Binder et al., 2005). The dual model predicts that abstract concepts are often understood as word descriptions, but not in terms of objects, events, and relations among them. (4) This model explains why language is acquired early in life, whereas cognition takes a lifetime. It also explains why children can acquire the entire hierarchy of language including abstract words without experience necessary for understanding them. (5) Since dynamic logic is the basic mechanism for learning language and cognitive representations, the dual model suggests that language representations become crisp after language is learned (5–7 years of age), however, cognitive representations may remain vague for much longer; the vagueness is exactly the meaning of “continuing learning,” this takes longer for more abstract and less used concepts. (6) The dual model gives mathematical description of the recursion mechanism (Perlovsky and Ilin, 2012). Whereas Hauser et al. (2002) postulate that recursion is a fundamental mechanism in cognition and language, the dual model suggests that recursion is not fundamental, hierarchy is a mechanism of recursion.

(7) Another mystery of human-cognition, not addressed by cognitive or language theories, is basic human irrationality. This has been widely discussed and experimentally demonstrated following discoveries of Tversky and Kahneman (1974), leading to the 2002 Nobel Prize. According to the dual hierarchy model, the “irrationality” originates from the dichotomy between cognition and language. Language is crisp and conscious while cognition might be vague and ignored when making decisions. Yet, collective wisdom accumulated in language may not be properly adapted to one’s personal circumstances, and therefore be irrational in a concrete situation. In the 12th century Maimonides wrote that Adam was expelled from paradise because he refused original thinking using his own cognitive models, but ate from the tree of knowledge and acquired collective wisdom of language (Levine and Perlovsky, 2008).

## Emotional prosody and its cognitive function

The dual model implies connections between language and cognitive representations, indicated by a wide horizontal arrow in Figure 1. These neural connections have to be developed and maintained. This requires motivation, in other words, emotions. These emotions must be in addition to utilitarian meanings of words, otherwise only practically useful words would be connected to their cognitive meanings. Also these emotions must “flow” from language to cognition, so that language is able to perform its cognitive function of guiding acquisition of cognitive representations, organizing experience according to cultural contents of language. These emotions therefore must be contained in language sounds, before cognitive contents are acquired.

This requirement of emotionality of language sounds is surprising and contradictory to assumed direction of evolution of language. Evolution of the language ability required rewiring of human brain in the direction of freeing vocalization from uncontrollable emotions (Deacon, 1997; Perlovsky, 2009b). Yet, the dual model requires that language sounds be emotional. Emotionality of human voice is most pronounced in songs (Perlovsky, 2010, 2012a,d, 2013b). Emotions of everyday speech are low, unless affectivity is specifically intended. We may not notice emotions in everyday “non-affective” speech. Nevertheless, this emotionality is important for developing the cognitive part of the dual model. If language is highly emotional, speakers are passionate about what they say, however, evolving new meanings might be slow, emotional ties of sounds to old meanings might be “too strong.” If language is low-emotional, new words are easy to create, however, motivation to develop the cognitive part of the dual model might be low, the real-world meaning of language sound might be lost. Cultural values might be lost as well. Indeed languages differ in how strong are emotional connections between sounds and meanings. This leads to cultural differences. Thus, the dual model leads to Emotional Sapir-Whorf Hypothesis (Perlovsky, 2007b, 2009b, 2012b). Strength of emotional connections between sound and meaning depends on language inflections. In particular, after English lost most of its inflections, it became a low emotional language, powerful for science and engineering. At the same time English is losing autonomous connections to cultural values that used to be partially inherent in language sounds. Fast change of cultural values during recent past is usually attributed to progress in thinking, whereas effects of change in emotionality of language sounds have not been noticed.

Emotional prosody can be important for overcoming cognitive dissonance. Cognitive dissonance is a discomfort due to holding contradictory cognitions (Festinger, 1957; Harmon-Jones et al., 2009). It is resolved by discarding contradictions. If a new word contradicts existing knowledge its meaning might be discarded. Emotional prosody as well as songs could be fundamental mechanisms that overcome cognitive dissonance and enable keeping new contradictory knowledge (Masataka and Perlovsky, 2012; Perlovsky, 2013a).

## Conclusion and experimental predictions

This article advances a hypothesis about functions of language and cognition in thinking, and possible model of their interactions. This is the only computable model explaining a number of mysteries about language and cognition and overcoming computational difficulties. It makes a number of predictions that could be experimentally tested, including the following: cognitive representations model the world, while language representations only model language; abstract cognitive representations can only be acquired due to language; abstract cognition is more clearly represented in language whereas cognitive representations may remain vague throughout life.
